# Risk Factors Affecting Survival Time of Breast Cancer Patients: The Case of Southwest Ethiopia

**DOI:** 10.34172/jrhs.2021.65

**Published:** 2021-10-12

**Authors:** Reta Habtamu Bacha, Yasin Negash Jabir, Anberbir Girma Asebot, Abebe Debu Liga

**Affiliations:** ^1^Department of Statistics, College of Natural Sciences, Jimma University, Jimma, Ethiopia; ^2^Department of Obstetrics and Gynecology, Institute of Health Sciences, Jimma University, Jimma, Ethiopia; ^3^Department of Statistics, College of Natural and Computational Sciences, Wolkite University, Wolkite, Ethiopia

**Keywords:** Acceleration Factor, Breast Cancer, Ethiopia, Parametric Shared Frailty Models

## Abstract

**Background:** Breast cancer is one of the non-communicable diseases and the main origin of the loss of life in the world. In Ethiopia, breast cancer is the second common cancer health problem for women. The main objective of this study was to identify the potential risk factors affecting the survival time of breast cancer patients in Southwest Ethiopia.

**Study design:** A retrospective study design.

**Methods:** The data were taken from the patients’ medical records that registered from January 1, 2015, to January 31, 2020. A retrospective study design was used in this study. Different shared frailty survival models were employed to analyze the dataset.

**Results:** Out of 642 recorded breast cancer patients, 447(69.6%) cases died during the study period, and 195 (30.4%) patients lost follow-up for unknown reasons. The median time to death for breast cancer patients was 10 months, and hospitals were used as a cluster effect. The result revealed that women with no smoking habit had about 3.35 times higher survival time than patients who had a smoking habit, and as breast cancer patients age increased, the survival time decreased by 0.99. Moreover, breast cancer patients in rural areas had about 0.14 times lower survival time, compared to breast cancer patients who were urban residents.

**Conclusions:** Age, place of residence, treatment taken, stage, histologic grade, tumor size, oral contraceptives, and smoking habits led to a shorter survival time. To reduce the burden of breast cancer, awareness should be given to the community.

## Introduction

Breast cancer is amongst the category of non-communicable diseases^1^. It is one of the root origins of loss of life, the highest commonly analyzed cancer, and the top cause of cancer death in women all over the world^2,3^. Globally, approximately 24.2% of new cancer cases and 15% of deaths occurred in 2018^4,5^. From this, 60% of deaths were observed in low- and middle-income countries^3,6^. By 2040, the projection of cancer is expected to be 28.4 million cases and 47% exceedance from 2020 with a larger increase in transitioning versus transitioned countries due to demographic variations though this may be further worsened by increasing risk factors associated with globalization and a rising economy^7^.

Breast cancer is held responsible for 28% of total cancer, and more than 24% of the incidence of breast cancer was recorded in Africa. The highest incidence rate of breast cancer was observed in North Africa, followed by East Africa^8^. Furthermore, Sub-Saharan African countries had the highest incidence rate with the highest age-standardized breast cancer death^2^. Nowadays, most countries of Africa face a double burden of cervical and breast cancer, which embodies the top cancer killer in women who are at least 30 years old^7^. Generally, in developed countries, breast cancer is a prominent source of loss of life among females^2^.

In Ethiopia, breast cancer is found to be the major cause of death^9^. Approximately, 22.6% of the breast cancer incidence and 17% of breast cancer deaths were observed^5,10^. Most of the women living in rural areas regularly pursue remedies from old-style therapists earlier than seeking help from the government health organization^11^.

In 2018, the estimated prevalence of breast cancer cases in Ethiopia was 13,987 with a crude incidence rate of 28.2 per 100,000 population, and it accounts for 33% of all cancer cases among women^10^. Studies showed that breast cancer is often diagnosed at an early stage, and patients have a good prognosis in developed countries. However, it is more often diagnosed at an advanced stage, and patients have low survival rates in developing countries, including Ethiopia^12^.

According to different studies in the literature, the risk factors associated with breast cancer are family history of cancer, place of residence, obesity, number of children, age, stage of breast cancer, menopause status, histological grade, region, and tumor size^11-17^. A study conducted by Tolosa et al.^18^ noted that the risk of breast cancer was higher among rural women, compared to those who lived in urban areas.

The main objective of this study was to identify risk factors affecting the survival time of breast cancer patients. In this study, time to death of breast cancer patients’ datasets were collected regularly until the patients died, fully recovered, or lost to follow-up due to breast cancer. Therefore, the data constitute survival data structures, and when the patient died, it was considered the main event of interest^19^. Accordingly, for such type of data, it is necessary to apply survival models. Kaplan Meier non-parametric survival models were used to estimate the survival time of patients^20^ or parametric models, such as the parametric shared frailty models, were applied in this regard^21-24^. In this study, the parametric shared frailty models were proposed to apply for modeling and inference of time to death of breast cancer patients.

## Methods

###  Data Source 

 A retrospective study was conducted in four randomly selected hospitals in Southwest Ethiopia. These four hospitals include Jimma Medical Center, Bedelle, Mizan-Aman, and Mettu Karl. The hospitals were selected by a simple random sampling method. The study population included all breast cancer patients who were registered at the selected hospitals with regular follow-up from January 1, 2015, to January 31, 2020. However, women who had cancer from another site, and those with insufficient information in the registration books were not eligible for the study. Therefore, women who were identified with confirmed breast cancer clinically and histologically, and those with full information in the registration books were eligible for inclusion. The starting point was when the women received treatment or were diagnosed at the hospital, and the ending point was when they died from breast cancer. A total of 642 cases were obtained using simple random sampling techniques with 95% confidence intervals.

###  Ethical Clearance 

 The Research Ethics Review Board of Jimma University, Jimma, Ethiopia, has provided ethical clearance for the study. The written formal cooperation letter was sent to the Jimma Medical Center, Bedelle, Mizan-Aman, and Mettu Karl where data were obtained. The study was conducted without individual informed consent or without including the name of the patients because it relied on retrospective data. The five-year card-based recorded data were obtained with their corresponding covariates.

###  Study variable 

The response variable is the time to death or survival time of breast cancer patients (which is measured in months) with an indicator of time of diagnosis and time to one of the event “death” that can be considered the event of interest in this study and coded as “1”. Moreover, lost to follow-up, dropped out, transferred to other hospitals with unknown reasons were considered censored and coded as “0”. To investigate the effect of risk factors on the survival time of breast cancer patients, factors known to affect the survival time regarding breast cancer were measured. These factors were classified as socio-demographic and clinical factors. The demographic factors were baseline age of patients, alcohol consumption categorized as yes or no, breastfeeding categorized as yes or no, smoking habits categorized as yes or no, and place of residence categorized as urban or rural. *The* clinical factors included treatment taken, stage of breast cancer which was according to the staging of cancer that was done using the American Joint Committee on Cancer 2002 system categorized as Stage I, II, III, and IV based on body mass index (BMI) kg/m^2^: obese (≥30.0), overweight (25.0-29.9), normal weight (18.5-24.9), and underweight (<18.5) ^25^, histological grade (well-differentiated, moderately-differentiated, and poorly-differentiated), tumor size (<2cm, 2-5cm, and >5cm), and family history (Yes or No) were risk factors considered in this study.

###  Statistical analysis 

The survival analysis was applied in this particular study. In survival analysis, there are often observations that need to be grouped together on the basis of the study center, city, and region. In this condition, the assumption of a homogenous population failed because of the unobserved covariates of individuals belonging to the same group. Since the assumption of homogeneity is failed, the appropriate way to handle unobserved heterogeneity is introducing the frailty term^21,26^. For determining the frailty effect, the most commonly and widely used distributions are Gamma and Inverse Gaussian, which act multiplicatively on the baseline hazard^23^. Because of its computational suitability, Gamma and inverse Gaussian were used as the frailty distribution for this study.

###  A shared gamma frailty model

The two-parameter gamma density function for the frailty term

$$v_{i}$$

with shape parameter *k *and scale parameter

λ

is given by

$$f_{v}(v_{i})\frac{k^{\lambda }v_{i}^{\lambda -1}e^{-kvi}}{\Gamma (\lambda )};\lambda >0,k>0$$

The Laplace transformed version of this density has the form^23^:


$$\eta (s)=\int_{0}^{\infty }\exp(-v_{i}s)f_{v}(v_{i})dv_{i}=k^{\lambda }{\left(s+k\right)}^{-\lambda }$$

The solution of the first and second partial derivatives of the Laplace function

η(.)

with respect to *s *solved to 0 gives the mean and variance of the frailty term,

$$\mu_{v}=E(v_{i})=\frac{k}{\lambda }$$

and

$$\sigma_{v}^{2}=\mathrm{var}(v_{i})=\frac{k}{\lambda^{2}}$$,

respectively.

###  The inverse Gaussian frailty model

The probability density function of an inverse Gaussian distributed frailty random variable

$$v_{i}$$

with parameters

μ>0

and

α>0

is given by^23^:


$$f_{v}(v_{i})=\frac{\alpha^{1/2}}{\sqrt{2\pi }}vi^{-3/2}\exp\left\{-\frac{\alpha }{2v_{i}\mu^{2}}{\left(v_{i}-\mu \right)}^{2}\right\},$$

where the Laplace transform of this function has a form


$$\eta (s)=\exp\left\{\frac{\alpha }{\mu }-{\left({\left(\frac{\alpha }{\mu }\right)}^{2}+2\alpha s\right)}^{1/2}\right\};s\geq 0$$

Then, the mean is

$$\mu_{v}=-\frac{\partial \eta (s)}{\partial s}\left|{}_{s=0}=\mu \right.$$

and variance

$$\sigma_{v}^{2}=-\frac{\partial \eta (s)}{\partial s}\left|{}_{s=0}\right.-{\left(\frac{\partial \eta (s)}{\partial s}\left|{}_{s=0}\right.\right)}^{2}=\frac{\mu^{3}}{\alpha }$$.

We set

$$\mu_{v}=1$$

for simplicity reason and we have that

$$\theta =\sigma_{v}^{2}=\frac{1}{\alpha }$$.

###  Parametric estimation 

There are various types of R-packages available. The ***parfm*** package^27^ was used for estimating the parameters of the parametric shared frailty models proposed in this study. The estimates and standard errors of the parameters of interest can be obtained from the *parfm* package.

###  Model comparison and diagnostics

Akaike Information Criterion (AIC)^28^ was used in this particular study, and the model with the smallest AIC value is considered a better fit^27^. After the model has been compared, it is crucial to check the effectiveness of the model in explaining the outcome. The identified accelerated failure time model should be linear and goes through the origin with the baseline distribution^20^.

## Results

Out of 642 breast cancer patients, 315 (70.8%) patients living in urban areas died, while 132 (67.0%) cases died in rural areas due to breast cancer. During the study period, out of 642 patients who had the smoking habit, 426 (70.6%) cases died, compared to 21 (53.8%) cases among non-smoker. Regarding tumor size, 83 (64.3%), 167 (71.4%), and 197 (70.6%) patients who had tumor size of below 2cm, between 2cm and 5cm, and above 5cm, died, respectively (Table 1).

**Table 1:** Characteristics of breast cancer patients from randomly selected governmental hospitals in the Southwest Ethiopia

**Variables**	**Censored count**		**Death count**		**Total count**		**Median** **(months)**
	**Number**	**Percent**	**Number**	**Percent**	**Number**	**Percent**	
Place of residency							
Urban	130	29.21	315	70.79	445	69.31	10.00
Rural	65	32.99	132	67.01	197	30.69	10.00
Smoking habit							
Yes	177	29.35	426	70.65	603	93.92	10.00
No	18	46.15	21	53.85	39	6.08	7.00
Tumor size (cm)							
<2	46	35.66	83	64.34	129	20.09	10.00
2-5	67	28.63	167	71.37	234	36.44	10.00
>5	82	29.39	197	70.61	279	43.47	10.00
Treatment taken							
Chemotherapy	49	22.89	165	77.11	214	33.33	10.00
Radiotherapy	60	37.03	102	62.97	162	25.23	11.00
Surgery	28	62.22	17	37.78	45	7.00	10.00
Hormonotherapy	19	37.25	32	62.75	51	7.94	10.00
Others	39	22.94	131	77.06	170	26.48	10.00
Stage							
I	3	11.54	23	88.46	26	4.05	11.00
II	49	23.33	161	76.67	210	32.71	10.00
III	72	31.44	157	68.56	229	35.67	10.00
IV	71	40.11	106	59.89	177	27.57	10.00
Body mass index (kg/m^2^)							
Underweight	52	32.09	110	67.91	162	25.23	10.00
Normal	50	27.47	132	72.53	182	28.35	11.00
Overweight	93	31.20	205	68.80	298	46.42	10.00
Histologic grade							
Well-differentiated	45	28.30	114	71.70	159	24.77	10.00
Moderately-differentiated	70	29.29	169	70.71	239	37.23	10.00
Poorly-differentiated	80	32.78	164	67.22	244	38.01	10.00
Alcohol consumption							
No	94	29.84	221	70.16	315	49.06	10.00
Yes	101	30.89	226	69.11	327	50.93	10.00
Family history of breast cancer							
No	90	32.14	190	67.86	280	43.61	10.00
Yes	105	29.01	257	70.99	362	56.39	10.00
Breastfeeding							
No	94	31.65	203	68.35	297	46.26	10.00
Yes	101	29.27	244	70.73	345	53.74	10.00
Oral contraceptives							
Not used	122	31.52	265	68.48	387	60.28	10.00
Used	73	28.62	182	71.38	255	39.72	11.00

Similarly, 165 (77.1%), 102 (63.0%), 17 (37.8%), 32 (62.7%), and 131 (77.1%) patients who took chemotherapy, radiotherapy, surgery, hormonotherapy, and other treatments died, respectively, and the rest were censored. In addition, from 177 (27.57%) breast cancer patients at stage IV, 106 (59.89%) cases died during the study period, whereas out of 229 (35.67%) breast cancer patients at stage III, 157 (68.56%) cases died, and the rest were censored (lost follow-up from the study with unknown reason). Furthermore, out of 298 (46.42%) overweight breast cancer patients, 205 (68.8%) of them died, while out of 162 (25.23%) underweight breast cancer patients, 110 (67.91%) cases died (Table 1).

The observed difference in survival experiences in different patient groups was also assessed using the Long-rank and Breslow test^20^. Table 2 shows a significant survival time difference in terms of smoking habit, treatment taken, stage of breast cancer patients, family history of breast cancer, and breastfeeding at a 5% significant level. Since the null hypothesis was rejected for these risk factors, post hoc analysis was conducted to perform pair-wise comparisons among the categories of factors.

**Table 2:** Comparison of survival time of breast cancer patients using the socio-demographic and clinical variables in the Southwest Ethiopia

**Variables**	**Mean survival time**	**Log Rank**	**P-value**	**Breslow**	***P*** **-value**	**df**
Place of residency		0.27	0.604	0.09	0.770	1
Urban	11.53					
Rural	11.46					
Smoking habit		10.62	0.001	15.35	0.000	1
Yes	11.73					
No	8.08					
Tumor size (cm)		1.43	0.489	1.07	0.585	2
<2	10.89					
2-5	11.41					
>5	11.86					
Treatment taken		20.32	0.004	17.42	0.016	4
Chemotherapy	11.73					
Radiotherapy	11.69					
Surgery	11.58					
Hormonotherapy	12.02					
Others	10.87					
Stage		11.84	0.008	8.50	0.037	3
I	11.38					
II	11.37					
III	11.36					
IV	11.87					
Obesity of patients		1.01	0.602	1.03	0.596	2
Underweight	10.98					
Normal	11.69					
Overweight	11.68					
Histologic grade		0.96	0.618	2.88	0.237	2
Well-differentiated	11.61					
Moderately-differentiated	11.34					
Poorly-differentiated	11.60					
Alcohol consumption		0.04	0.833	1.01	0.315	1
No	11.62					
Yes	11.39					
Family history of breast cancer		6.33	0.012	5.09	0.024	1
No	12.02					
Yes	11.10					
Breastfeeding		6.33	0.012	5.09	0.024	1
No	11.23					
Yes	11.74					
Oral contraceptives		1.12	0.289	2.33	0.127	1
Not used	11.18					
Used	12.00					

The survival curves of breast cancer patients were different regarding smoking habit, treatment taken, stage of breast cancer, family history of breast cancer, and breastfeeding. However, place of residence, tumor size, obesity, histologic grade, alcohol consumption, and oral contraceptives did not show a clear difference (Table 2)

Furthermore, Table 3 indicates the summary status of breast cancer patients in Southwest Ethiopia. From this summary, the median follow-up time was 10 months for patients that were censored, and about 75% of the patients had 14 months of follow-up (upper quartile). The median time to death by breast cancer was obtained at 10 months.

**Table 3:** Summary status of breast cancer patients and months of follow-up time in the Southwest Ethiopia

**Status of patients**	**Number**	**Percent**	**Mean**	**SD**	**Median**	**Lower quartile**	**Upper quartile**
Censored	195	30.4	11.50	4.76	10.00	8.00	14.00
Dead	447	69.6	11.51	4.59	10.00	8.00	14.00

###  Comparison of Models with Akaike Information Criterion

This study was conducted by considering the four-baseline hazard functions, such as Weibull, Log-logistic, Log-normal, and exponential, as well as two frailty distributions, such as Gamma and Inverse-Gaussian. Accordingly, the Gamma and Inverse-Gaussian shared frailty model was fitted to select the best model for this study using hospitals as random (frailty). The AIC value of the Log-normal-inverse Gaussian model was 2838.15, and the minimum among the other AIC values of the models indicated that it was the most efficient model to describe the breast cancer dataset using parametric frailty models (Table 4).

**Table 4:** Comparison of models with Akaike Information Criterion

** Baseline**	**Frailty**	
	**Gamma**	**Inverse- Gaussian**
Weibull	2897.56	2887.14
Exponential	2878.14	2868.54
Log-logistic	2867.56	2868.59
Log-normal	2858.15	2838.15

Based on the result of the Log-normal-inverse Gaussian shared frailty model, the age, place of residence, treatment taken, stage, histological grade, tumor size, smoking habits, and oral contraceptives were significant. However, obesity, family history of breast cancer, and breastfeeding were not significant variables (Table 5).

**Table 5:** Multivariable Log-normal-inverse Gaussian shared frailty model

**Variables**	**Coef.**	**SE**	ϕ** (95% CI)**	***P*** **-value**
Age (yr)	-0.01	0.01	0.99 (0.98, 0.99)	0.024
**Place of residence**				
Urban	Ref.			
Rural	-1.97	0.11	0.14 (0.11, 0.17)	0.006
**Treatment taken**				
Chemotherapy	Ref.			
Radiotherapy	-0.29	0.13	0.79 (0.58, 0.97)	0.027
Surgery	-0.92	0.30	0.40 (0.22, 0.72)	0.002
Hormone-therapy	-0.58	0.21	0.56 (0.37, 0.85)	0.006
Others	0.13	0.122	1.14 (0.90, 1.45)	0.278
**Stage**				
Stage I	Ref.			
Stage II	-0.91	0.19	0.40 (0.28, 0.58)	0.001
Stage III	-0.95	0.19	0.39 (0.27, 0.56)	0.001
Stage IV	-1.15	0.19	0.32 (0.22, 0.46)	0.001
**Obesity**				
Underweight	Ref.			
Normal	-0.22	0.13	0.80 (0.62, 1.04)	0.096
Overweight	-0.14	0.12	0.87 (0.68, 1.10)	0.250
**Histologic grade (differentiation)**		
Well	Ref.			
Moderate	-0.18	0.12	0.84 (0.66, 1.06)	0.148
Poor	-0.44	0.13	0.64 (0.50, 0.83)	0.001
**Alcohol consumption**				
No	Ref.			
Yes	-0.04	0.10	0.97 (0.79, 1.18)	0.736
**Family history of breast cancer**			
No	Ref.			
With	-0.04	0.12	0.99 (0.82, 1.21)	0.971
**Breastfeeding**				
No	Ref.			
Yes	-0.18	0.10	0.84 (0.69, 1.02)	0.083
**Tumor size (cm)**				
<2	Ref.			
2-5	-0.36	0.11	0.69 (0.56, 0.86)	0.004
>5	-0.54	0.12	0.59 (0.46, 0.75)	0.025
**Smoking habit**				
Yes	Ref.			
No	1.21	0.23	3.35 (2.12, 5.27)	0.001
**Oral contraceptives**				
Not used	Ref.			
Used	-0.25	0.11	0.78 (0.64, 0.96)	0.021

τ= 0.333; θ= 0.998; δ = 0.214; Likelihood ratio test=16.8; df= 11.1, P=0.004

In the shared frailty model, the most important thing is the interpretation of the acceleration factor. It can be interpreted as if 1 is not included in the acceleration confidence interval, then the factors are statistically significant; otherwise, they are not significant. An acceleration factor (*ϕ*) greater than 1 specifies prolonging the time to death. From Log-normal-inverse Gaussian shared frailty model, it was found that the increase of age (ϕ*=*0.99; 95% CI: 0.98-0.99) led to a decrease in the survival time of breast cancer patients. The acceleration factor for the rural residents was 0.14. This implies that rural residents had a shorter time to death, compared to breast cancer patients who were urban residents.

 Patients at stage IV had an acceleration factor of 0.32 (95% CI: 0.22-0.46) which indicated that patients in stage I had a longer survival time, compared to breast cancer patients at stage IV. Moreover, the patients who had no smoking habit had longer survival time than those who had a smoking habit (ϕ=3.35; 95%CI: 2.12-5.27). In addition, patients who used oral contraceptives (ϕ=0.78; 95% CI: 0.64-0.96) had a lower survival time, compared to those who used no oral contraceptives. The acceleration factor and its 95% CI for poorly-differentiated histologic grade were 0.64, as well as 0.50 and 0.83, respectively. This indicates that poorly-differentiated histologic grades for breast cancer patients had lower survival time than well-differentiated histologic grades for breast cancer patients. The estimate of the shape parameters in the Log-normal-inverse Gaussian shared frailty model is (δ=0.214). The heterogeneity in the population of the treatment center which is used as a cluster is estimated by our selected model at θ=0.998 (P=0.004), and the dependence within clusters was about τ=33.3%. There were differences in the death rate of patients in different hospitals of Southwest Ethiopia.

###  Model Diagnostics

**Figure 1:**
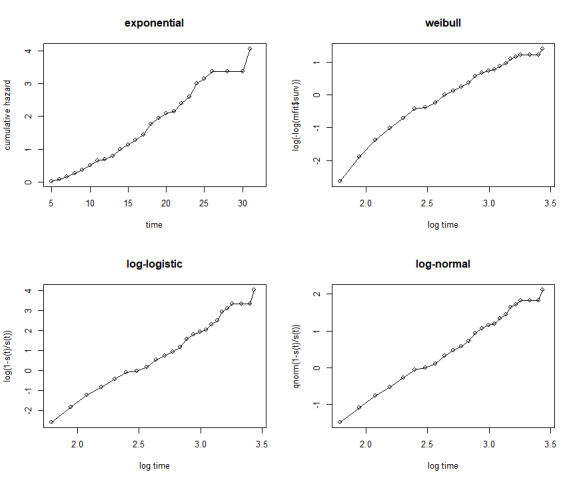
Graphs of exponential, Weibull, Log-logistic, and Log-normal baseline distributions for time to death of breast cancer patients

If the plot of the Weibull, Log-normal, Log-logistic, or exponential are linear, the given baseline distribution is appropriate for the given dataset ^29,30^. Accordingly, their respective plots are given in Figure 1, and the plots for the Log-normal baseline distribution make the straight lines better than Weibull, exponential, and Log-logistic baseline distributions.

## Discussion

Different accelerated failure time models were applied to analyze the datasets since there was heterogeneity in the population of the treatment center (hospitals), which was used as a cluster effect. Based on the AIC value, it was found that Log-normal-inverse Gaussian was the best fit for the datasets. In addition, it was observed that as the stage of breast cancer increased, the survival rate of breast cancer patients decreased. This result is consistent with the findings of the studies conducted by Hoang P et al. and Mensah A et al. ^31,32^. Based on the results, as the ages of breast cancer patients increase, the survival time decreases. This finding is also in line with the results of a study performed by Allemani C et al.^33^ This evidence also strengthens the decision made by the AIC values that Log-normal baseline distribution is appropriate for the given dataset.

The findings of this study revealed that breast cancer patients who had a tumor size of between 2cm and 5cm and above 5cm had shorter survival time, compared to those who have tumor size below 2cm (which was used as a reference category). This result was similar to the findings of a previous study carried out in Ghana^32^.

In this study, breast cancer patients with poorly-differentiated histologic grades had lower survival time, compared to well-differentiated histologic grades (reference group). This finding is consistent with the results of the studies conducted by Baghestani AR et al. and Alotaibi RM et al.^16,17^. The result also indicated that the breast cancer patients who lived in rural areas had lower survival times than those who lived in urban areas. The result is verified by the studies carried out by Tolessa L et al. and Balekouzou A et al.^18,34^. This is due to less awareness about health-related issues in rural areas. Moreover, even those who had awareness might not have had access to health centers due to limited resources in the hospitals.

The study revealed that breast cancer patients who were oral contraceptive users had lower survival time, compared to those who did not use oral contraceptives. However, this result is not consistent with the findings of a study performed by Brinton LA et al. ^35^, which indicated that oral contraceptive use did not seem to increase the risk of breast cancer. However, oral contraceptive use before a first full-term pregnancy or for more than five years can modify the development of breast cancer.

In this study, breastfeeding, obesity, and family history were not found to be the risk factors that affect the survival time of breast cancer patients. However, different studies reported that women with a family history of cancer, and those who were obese were more likely to be affected with breast cancer ^18,36,37,38^. Smoking habit is an important risk factor significantly influencing breast cancer in this study. Women who did not smoke had prolonged their survival time three times more than those who did not smoke. This result is consistent with the findings of a study conducted by Catsburg C et al.^39^. They found a strong association between smoking and breast cancer; moreover, they noted that the timing of cigarette exposure was also important in this regard.

###  Strength and Limitations of Study 

 Regarding the strength of the study, different shared frailty survival models were applied to identify the risk factors affecting the survival time of breast cancer to handle heterogeneity in hospitals. Second, an appropriate sampling design was applied to collect the datasets. However, in this study, most clinical variables were not included, and they were only limited to the variables mentioned in the methodological part since they were not with full information. This in turn might affect our conclusions.

## Conclusions

 Factors, such as age, place of residence, treatment taken, stage, histologic grade, tumor size, smoking habit, and oral contraceptives were significantly influencing breast cancer patients. The breast cancer patients with higher age, smoking habit, oral contraceptive use, poorly-differentiated histologic grade, stage IV of breast cancer, and rural residency had shorter survival time. On the other hand, early stage (stage I), well-differentiated histological grade, urban residency, lack of oral contraceptive use, and no smoking habit had prolonged survival time of breast cancer patients, compared to others. Awareness should be given to the community to reduce the burden of breast cancer.

## Acknowledgments

 Firstly, all the authors would like to thank Jimma University, College of Natural Sciences Research and post-graduating office for providing financial support for this study and would like to thank Jimma Medical Center, Bedelle, Mizan-Aman, and Mettu Karl hospitals in Southwest Ethiopia for providing data on breast cancer patients.

## Conflict of Interests

 The authors declare that they have no conflict of interest.

## Funding

 This study was financially supported by the College of Natural Sciences, Jimma University, Jimma, Ethiopia. The supporting bodies had no role in data collection, analysis, and preparation of the manuscript or in the decision to publish.

## Authors’ contributions’

 RHB contributed to the study concept, design, and drafted the manuscript. YNJ participated in the data collection and drafted the manuscript. AGA reviewed the manuscript. ABL participated in data collection and drafted the manuscript. All authors approved the final manuscript.

 Highlights

- The median time to death by breast cancer was 10 months.
- The log-rank and Breslow test were applied to identify whether there is a survival difference in the categories of the risk factors affecting the survival time of breast cancer patients in Southwest Ethiopia.
- The Log-Normal baseline distribution with Inverse Gaussian frailty is the best fit for breast cancer patient datasets.
- The acceleration factor measures the risk effect of factors on the event of interest and was computed manually for significant risk factors.
